# Management of lentiginous melanoma with imiquimod assessed by reflectance confocal microscopy

**DOI:** 10.1002/ski2.212

**Published:** 2023-01-14

**Authors:** Joseph Han, Hansen Tai, Dina Poplausky, Jade Young, Rishab Revankar, Peter Baek, Samantha Walsh, Nicholas Gulati

**Affiliations:** ^1^ Department of Dermatology Icahn School of Medicine at Mount Sinai New York New York USA; ^2^ Levy Library Icahn School of Medicine at Mount Sinai New York New York USA

## ETHICS STATEMENT

Not applicable.

To the Editor

1

Lentiginous melanoma (LM) has poorly defined margins and may be subject to inaccurate assessment by traditional methods, including clinical evaluation and dermoscopy.^1^ Imiquimod, a topical immunomodulatory agent, has been used as an alternative to surgical treatment for LM, but concern for incomplete tumour clearance limits its widespread adoption. Reflectance confocal microscopy (RCM) is a high‐resolution, non‐invasive imaging technique and a reliable diagnostic tool to identify LM, with a sensitivity of 85% and specificity of 76%.^2^ Given LM often presents on cosmetically sensitive regions such as the face, non‐invasive therapy and monitoring techniques, such as imiquimod and RCM, respectively, may be preferred.

We systematically reviewed the literature on LM treated with imiquimod and assessed by RCM. We identified 10 relevant publications and graded the evidence using the Joanna Briggs Institute (JBI) critical appraisal tool (Table 1, Supplementary Figure S1). Across all studies, there was a total of 168 participants, of which 114 were female and 54 were male. Participants' ages ranged from 33 to 90. Facial LM was the most common type of LM evaluated in all publications reviewed. In 7/10 publications, a treatment regimen of imiquimod 5% daily, five times per week was utilised. The average length of imiquimod treatment in the evaluated studies was approximately 10 weeks.

**TABLE 1:** Characteristics and outcomes of included studies

Author	Study design	Participant count^a^	RCM application relative to imiquimod treatment	Treatment regimen	Comparator for margin evaluation	Quality of evidence^b^	Initial outcome (imiquimod response)^c^	RCM concordance with histopathology	Long‐term outcome (tumour recurrence)	Treatment for recurrent/residual LM
Alarcon et al. (2014)^3^	Prospective single‐arm cohort	20	Pre‐treatment, 12 weeks post‐treatment, 12 months post‐treatment	Imiquimod 5% daily, 5x/week for 8 weeks	Histopathology	9	15/20 of patients had complete clearance	RCM was concordant with histopathology in 19/20 patients. No statistically significant difference in sensitivity (*p* = 1) or specificity (*p* > 0.05) between RCM and histopathology. There were no false negatives by RCM.	15/15 patients had no recurrence at mean follow up of 34 months	‐
Alani et al. (2016)^4^	Case report	1	Pre‐treatment, 9 months post‐treatment	Imiquimod 5% 5x/week for 6 weeks	Histopathology	10	1/1 patient had complete clearance	RCM was concordant with histopathology in 1/1 patient	‐	‐
Brand et al. (2019)^5^	Prospective single‐arm cohort	34	Pre‐treatment, post‐treatment, duration unspecified	Imiquimod 5% 1–2x per day for 8–108 days	Clinical evaluation, dermoscopy, histopathology	9	33/34 patients had therapeutic response	RCM strongly correlated with histopathology ‐ *r* = 0.7335, *p* = 0.0001	27/34 patients had no recurrence, 7/34 had recurrence (mean time 3.07 years)	Imiquimod (*n* = 2) and surgery (*n* = 5)
Coco et al. (2021)^6^	Case series	3	Pre‐treatment, 6 weeks post‐treatment, 18 months post‐treatment	Imiquimod 5% 3–5x/week for 6–18 weeks	Clinical evaluation, dermoscopy, histopathology	7	Two‐thirds patients had complete clearance, one‐third had a partial response	RCM was concordant with histopathology in 2/2 patients that had biopsies performed, one patient was lost to follow up	2/2 patients had no recurrence at 18 months follow up, excluding patient lost to follow up	‐
Guitera et al. (2013)^1^	Retrospective single‐arm cohort	7	Pre‐treatment, 7–66 months post‐treatment	Imiquimod 5%, 5x/week for 12 weeks	Clinical evaluation, dermoscopy, histopathology	10	‐	RCM was concordant with histopathology in 7/7 patients	6/7 patients had no recurrence, 1/7 patients had recurrence at 1 year (tumour had previously recurred five times), and subsequently cleared at 40 months	Imiquimod
Guitera et al. (2014)^7^	Retrospective cohort	28	Pre‐treatment, post‐treatment	Imiquimod 5x/week for 12 weeks	Clinical evaluation, dermoscopy, histopathology	8	25/28 patients had complete clearance (19 by RCM, 6 by histopathology)	RCM was concordant with histopathology for LM monitoring with a specificity of 94% and sensitivity of 100%	‐	Imiquimod or surgery
Hibler et al. (2017)^8^	Case report	1	Post‐treatment/pre‐surgery, post‐surgery	Imiquimod 5%	Histopathology	7	0/1 patients had complete clearance	RCM was concordant with histopathology in 1/1 patient.	‐	Staged excision
Kai et al. (2016)^9^	Case series	18	5.6–10.1 years post‐treatment	Imiquimod 3–5x/week for 4–8 weeks	Clinical evaluation, histopathology	10	27/40 patients had complete clearance	RCM and histopathology used independently at different time points	18/27 patients had no recurrence at >5 years follow up	Mohs surgery
Nadiminti et al. (2010)^10^	Case series	2	Pre‐treatment, 3–6 months post‐treatment	Imiquimod daily for 12–27 weeks	Clinical evaluation, dermoscopy, histopathology	8	Half patients had complete clearance	RCM was concordant with histopathology in 2/2 patients	1/1 patient had no recurrence at 2 years follow up	Staged excision
Soenen et al. (2022)^11^	Placebo‐controlled, randomized study	21	Pre‐treatment, 1‐month post‐treatment	Imiquimod 5x/week for 4 weeks	Clinical evaluation, histopathology	8	13/21 patients had complete clearance	RCM was concordant with histopathology in 16/21 patients treated with imiquimod. Discrepancy was not statistically significant (*p* = 0.8). Second blinded dermatopathologist found RCM was concordant with histopathology in 19/21 patients.	‐	‐

Abbreviations: LM, lentiginous melanoma; RCM, reflectance confocal microscopy.

^a^ Participant count includes patients treated with imiquimod and evaluated by RCM.

^b^ Quality of evidence was assessed on a 10‐point scale using the Joanna Briggs Institute (JBI) critical appraisal tool specific to each article type. Scores out of 100% were calculated for all relevant fields for each article, and scores were associated with an evidence grade 1–10 (0%–10% corresponding to grade 1%, 11%–20% corresponding to grade 2, and so on).

^c^ Imiquimod response (tumour clearance) evaluated by histopathology unless otherwise specified.

Studies reproducibly demonstrated imiquimod as a potential treatment alternative to traditional surgical excision. Furthermore, RCM margin evaluation had high concordance with gold standard histopathology (Table 1). For the studies that specified evaluation time points, RCM assessment was performed four or more weeks after treatment with imiquimod. One prospective study (*n* = 20) reported LM histopathologic clearance with imiquimod in 75% of patients. RCM identified 93% of the responders with no false negatives (compared to histopathology) at 12 weeks and 12 months after treatment. Responders continued to have histopathological clearance for a mean of 34 months^3^

Several retrospective studies demonstrated imiquimod to be an adequate treatment for LM with concurrent RCM monitoring. In one study (*n* = 18) evaluating 5‐year recurrence rates of LM after imiquimod treatment, all 18 patients had histopathologically clear margins 3 months post‐treatment. Long‐term tumour clearance (mean follow‐up 7.4 years) measured by RCM revealed no evidence of recurrence in any patient.^9^ In another retrospective study (*n* = 34), 33/34 patients demonstrated initial therapeutic response with imiquimod as measured by RCM and histopathology. Seven patients had LM recurrence within a mean of 3.07 years. RCM measurements significantly correlated with histopathology results (*r* = 0.7335, *p* = 0.0001).^5^ Another retrospective study detected recurrence in 1/7 patients treated with imiquimod at 1‐year follow‐up (tumour had previously recurred five times). This patient was treated with a subsequent course of imiquimod, and no recurrence was detected via RCM evaluation at 40 months following this second treatment.^1^ A subsequent retrospective study (*n* = 28) demonstrated complete clearance in 25/28 participants treated with imiquimod. In comparison to histopathological evaluation for tumour recurrence, RCM had a specificity of 94% and a sensitivity of 100%.^7^

In a case series of three participants treated with imiquimod for LM, two achieved complete clearance and had no recurrence after an 18‐month follow‐up period, evaluated by RCM and histopathology concordantly. One patient achieved partial clearance at initial evaluation by RCM, but was lost to follow‐up.^6^ An additional case series included two patients with LM treated with imiquimod. One patient achieved complete clearance after 3 months of therapy and remained disease‐free 2 years after treatment. The other patient failed 7 months of treatment and required surgical excision. RCM was concordant with histopathological evaluation in both cases.^10^

A randomized controlled trial evaluated imiquimod as a neoadjunvant treatment prior to surgery. Researchers compared the pre‐surgical treatment of LM with imiquimod versus placebo. Prior to surgery, pre‐determined RCM features, such as non‐edged dermal papillae, were significantly decreased in the imiquimod arm (*n* = 21) versus placebo (*n* = 19) after 1 month of treatment (*p* < 0.001). RCM was concordant with histology in 19 of 21 patients (90%) with no false negatives.^11^

Current data demonstrate that imiquimod can be effectively utilised in the treatment of LM. This review is limited by varying follow‐up times for long‐term tumour recurrence monitoring. Prior data have shown that the median time to LM recurrence after treatment is 3.8 years^12^ Two of the included publications had a follow‐up period greater than 3.8 years. In these studies, a tumour recurrence was detected in 10/34 patients. Given that, imiquimod alone may not be sufficient for complete LM clearance and may be more suitable as an adjunctive therapy. Larger prospective studies are warranted to explore the limitations of and an appropriate clinical scenario for imiquimod as monotherapy for LM treatment. RCM demonstrated margin evaluation concordance or significant positive correlation with histopathology in 9/10 publications. Of particular clinical importance, in the 8 studies that reported concordance rates, there were no false negatives when RCM was used to evaluate for residual LM following imiquimod treatment. This supports its potential use as a non‐invasive and reliable method to monitor imiquimod treatment of LM.

## CONFLICTS OF INTEREST

The authors declare that there is no conflict of interest.

## AUTHOR CONTRIBUTIONS

**Joseph Han**: Conceptualization (Lead); Data curation (Lead); Formal analysis (Lead); Investigation (Lead); Methodology (Lead); Project administration (Lead); Validation (Lead); Visualization (Lead); Writing – original draft (Lead); Writing – review & editing; Lead. **Hansen Tai**: Conceptualization (Lead); Data curation (Lead); Formal analysis (Lead); Investigation (Lead); Methodology (Lead); Project administration (Lead); Validation (Lead); Visualization (Lead); Writing – original draft (Lead); Writing – review & editing (Lead). **Dina Poplausky**: Data curation (Equal); Formal analysis (Equal); Investigation (Equal); Methodology (Equal); Project administration (Equal); Validation (Equal); Visualization (Equal); Writing – original draft (Equal); Writing – review & editing (Equal). **Jade Young**: Data curation (Equal); Formal analysis (Equal); Investigation (Equal); Methodology (Equal); Project administration (Equal); Validation (Equal); Visualization (Equal); Writing – original draft (Equal); Writing – review & editing; Equal. **Rishab Revankar**: Data curation (Equal); Investigation (Equal). **Peter Baek**: Data curation (Equal); Investigation (Equal); **Samantha Walsh**: Data curation (Lead); Methodology (Lead); **Nicholas Gulati**: Conceptualization (Lead); Methodology (Lead); Project administration (Lead); Supervision (Lead); Writing – review & editing (Lead).

## FUNDING INFORMATION

This article received no specific grant from any funding agency in the public, commercial, or not‐for‐profit sectors.

## Supporting information

Supplementary Figure S1

## Data Availability

The data that supports the findings of this study are available in the supplementary material of this article.
