# Supplementary figures and images for: Management of lentiginous melanoma with imiquimod assessed by reflectance confocal microscopy
Source: Skin Health Dis. 2023 Jan 14;3(2):e212. doi: 10.1002/ski2.212 (PMC10066750; doi:10.1002/ski2.212)

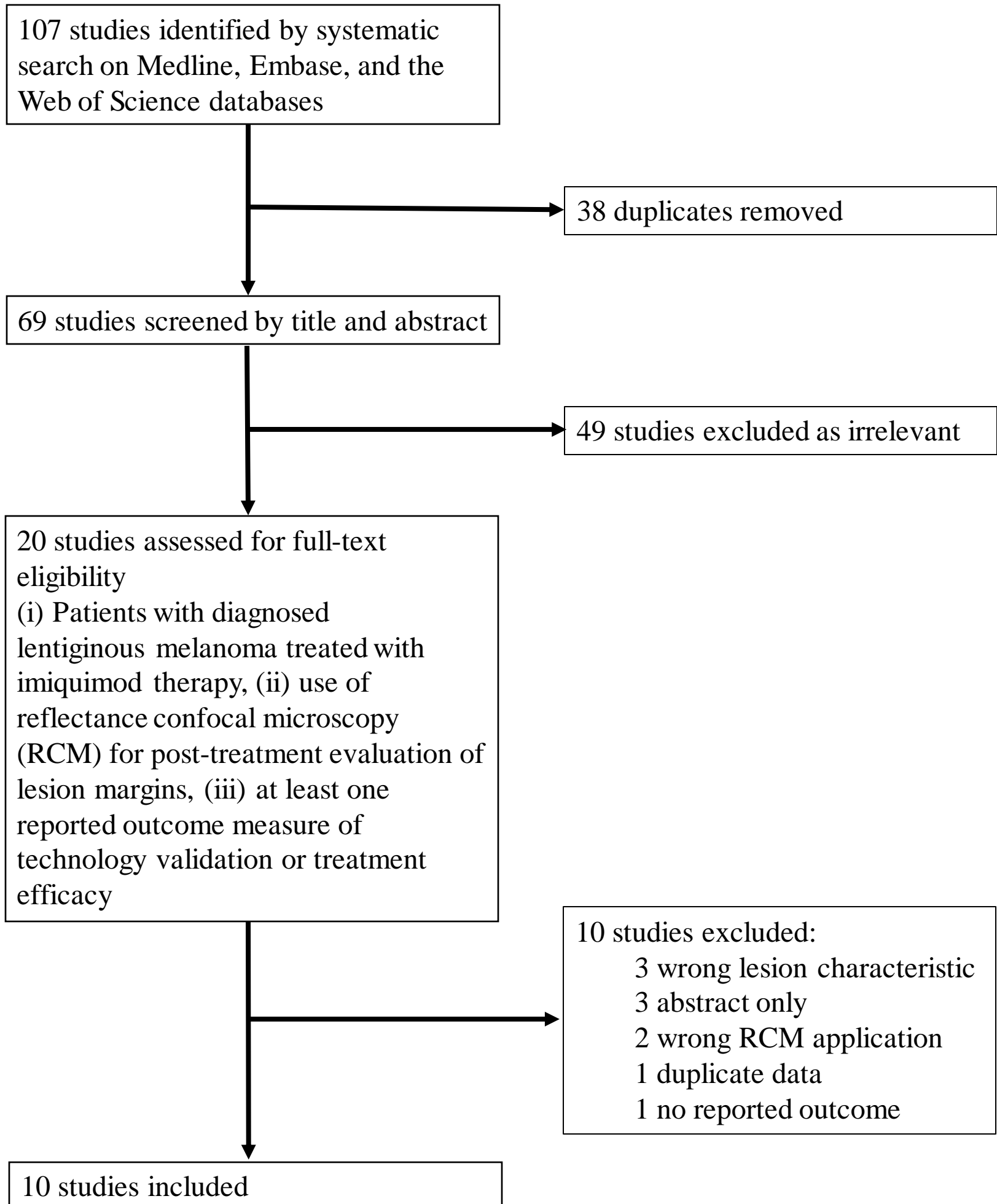

Supplement: Supplementary file 1 — Supplementary Figure S1 [file SKI2-3-e212-s001.pdf]
